# Dr. Charles Harrington

**Published:** 1908-11

**Authors:** 


### OBITUARY

---

DR. CHARLES HARRINGTON, of Boston, one of the most distinguished sanitarians in America, died at Lynton, England, September 11, 1908, aged 52 years. His death took place while he was on vacation, was sudden, and presumably caused by heart disease. He was an ornament to his profession and his death is a distinct loss to scientific, especially sanitary, medicine the world over.

